# Early mutational signatures and transmissibility of SARS-CoV-2 Gamma and Lambda variants in Chile

**DOI:** 10.1038/s41598-024-66885-2

**Published:** 2024-07-11

**Authors:** Karen Y. Oróstica, Sebastian B. Mohr, Jonas Dehning, Simon Bauer, David Medina-Ortiz, Emil N. Iftekhar, Karen Mujica, Paulo C. Covarrubias, Soledad Ulloa, Andrés E. Castillo, Anamaría Daza-Sánchez, Ricardo A. Verdugo, Jorge Fernández, Álvaro Olivera-Nappa, Viola Priesemann, Seba Contreras

**Affiliations:** 1 Facultad de Medicina, Universidad de Talca, Talca, Chile; 2 Max Planck Institute for Dynamics and Self-Organization, Göttingen, Germany; 3 Institute for the Dynamics of Complex Systems, University of Göttingen, Göttingen, Germany; 4 Departamento de Ingeniería en Computación, Universidad de Magallanes, Punta Arenas, Chile; 5 Sub Department of Molecular Genetics, Institute of Public Health of Chile (ISP), Santiago, Chile; 6 Centre for Biotechnology and Bioengineering, Universidad de Chile, Santiago, Chile; 7 Departamento de Oncología Básico-Clínica, Facultad de Medicina, Universidad de Chile, Santiago, Chile; 8 Department of Chemical Engineering, Biotechnology and Materials, Universidad de Chile, Santiago, Chile

**Keywords:** Epidemiology, Statistics, Viral infection, Mutation, Applied mathematics

## Abstract

Genomic surveillance (GS) programmes were crucial in identifying and quantifying the mutating patterns of SARS-CoV-2 during the COVID-19 pandemic. In this work, we develop a Bayesian framework to quantify the relative transmissibility of different variants tailored for regions with limited GS. We use it to study the relative transmissibility of SARS-CoV-2 variants in Chile. Among the 3443 SARS-CoV-2 genomes collected between January and June 2021, where sampling was designed to be representative, the Gamma (P.1), Lambda (C.37), Alpha (B.1.1.7), B.1.1.348, and B.1.1 lineages were predominant. We found that Lambda and Gamma variants’ reproduction numbers were 5% (95% CI: [1%, 14%]) and 16% (95% CI: [11%, 21%]) larger than Alpha’s, respectively. Besides, we observed a systematic mutation enrichment in the Spike gene for all circulating variants, which strongly correlated with variants’ transmissibility during the studied period (r = 0.93, p-value = 0.025). We also characterised the mutational signatures of local samples and their evolution over time and with the progress of vaccination, comparing them with those of samples collected in other regions worldwide. Altogether, our work provides a reliable method for quantifying variant transmissibility under subsampling and emphasises the importance of continuous genomic surveillance.

## Introduction

The COVID-19 pandemic was marked by the high rate at which new SARS-CoV-2 variants emerged^1,2^. This high mutational rate could be due to the natural properties of the virus^3,4^ and the surge in COVID-19 incidence that followed the lifting of major non-pharmaceutical interventions (NPIs) in some countries^5–8^. Genomic surveillance (GS) has unveiled the rapid evolution of SARS-CoV-2 and signalled the emergence of variants with increased transmissibility and partial immune escape (e.g., those labelled as Variants of Concern VoC)^9–12^, thereby supporting evidence-based decisions in epidemic management^9,11,13–16^. In the context of the COVID-19 pandemic, GS consisted of sequencing specific samples that tested positive for COVID-19 (i.e., determining their genome or parts of it). As of May 2024, GS programs worldwide have reported more than 16.7 million SARS-CoV-2 genomes to the GISAID database^12,17^, where they are collected and shared. At a country scale, the quality of the information gathered by GS depends on the number of samples analysed and the protocol used to select them^11,15^. The number of samples that can be analysed, however, responds to an economic trade-off, where the costs associated with GS remain prohibitive for low and middle-income countries^18–23^. For example, in Chile, despite the governmental and private investments in GS, the sequencing rate has been around 400 samples per week (i.e., 20 samples per million inhabs.), at least two orders of magnitude smaller than European countries such as Denmark, Germany, and the UK^9,15,24^. Selecting which samples should be sequenced in these settings is fundamental for avoiding biases and misleading results.

The spread of COVID-19 in Chile has been remarkably heterogeneous, not only because of its geography and sparse urbanisation but also because of the pronounced social inequalities^25–32^. Although the Chilean government deployed an ambitious vaccination program as soon as the vaccines became available^33–36^, containment of local outbreaks was still challenged by the early lifting of NPIs due to economic pressures^37^, reporting delays^38^, inefficient contact tracing^39^, and the comparatively low protection against infection granted by the predominant vaccine^40^. Furthermore, the partial isolation of some areas of Chile and the fast connections to Santiago, the capital city, further favours the spread of locally generated variants^41^ or the insertion of new lineages in zones where there were no cases. The above highlights the importance of optimising available GS resources to alert policymakers about locally emerging threats, such as the Lambda lineage^11,42–44^.

Here, we quantify the contribution of different SARS-CoV-2 variants to the spread of COVID-19 in Chile, a country that has a limited but consistent ability to conduct GS. Among the 3443 genomes collected from January to June 2021, the Gamma (P.1), Lambda (C.37), Alpha (B.1.1.7), B.1.1.348, and B.1.1 lineages were the most common. We ensured that the samples were representative of the population. To that end, we created a Bayesian framework to assess the relative transmissibility of variants specifically designed for regions with limited GS resources (i.e., regions under subsampling). This framework can be adapted for use in other areas, enhancing the effectiveness of surveillance programs. In addition to finding significant differences in the transmissibility of co-circulating variants, we observed changes over time in the mutational signatures of the sequenced samples. This suggests the presence of a selective pressure leading to lineage differentiation and emphasises the importance of studying the spread of these variants at a regional level.

## Methods overview

We studied surveillance data of 3443 samples collected between January and June 2021 from different Chilean regions in hospitals belonging to the Chilean influenza surveillance network. All samples must have tested positive in an RT-qPCR SARS-CoV-2 test with a Ct value lower than 25 and were sent to the Chilean Public Health Institute (ISP) in Santiago for sequencing under a strict cold transportation chain. Whole SARS-CoV-2 genome sequences were obtained using a MiSeq (Illumina) platform with a 300-cycle (total) reagent kit. We assessed sequencing quality with the FastQC program, v0.11.8, and used the IRMA (v0.9.3) and MAFFT (v7.458) software to assemble and align the genomes respectively^45,46^. The lineage to which each sample belongs was determined using Pangolin v3.1.5^47^. Then, we defined the most prevalent lineages through the frequency of observation per epidemiological week, thus selecting the lineages with a frequency equal to or greater than 20%. We limit our analysis window to samples collected from January and June 2021, as after this point, the representativeness of the sampling protocol for GS was compromised; samples suspected to belong to the Delta lineage (B.1.617.2) were prioritised for sequencing to achieve other public health goals^48,49^.

The Chilean Ministry of Health coordinates the national influenza/SARS-CoV-2 surveillance network of hospitals and care centres, thus having the responsibility of collecting, selecting, and choosing which samples need to be sequenced by the ISP. Once these samples are sequenced, they are promptly shared with the international public repository GISAID, making them available for this and other scientific studies. Therefore, given that the data we used for this study was of an open domain, no patient consent was required.

Our Bayesian model simulates the spread of each variant separately using a discrete renewal process^50–53^. In our model, COVID-19 spreads with an inferred time-dependent effective reproduction number $R_{\text {eff}, t}$^54^, where the contribution of each variant to $R_{\text {eff}, t}$ is modulated by a time-invariant factor $f_{\text {variant}}$. This factor accounts for the relative transmissibility of the variants. We use the Alpha variant as reference (i.e., $f_\mathrm{Alpha} = 1$), as its transmissibility has been accurately quantified in settings where it was the sole circulating variant^55^; Knowing the base reproduction number of Alpha enables the estimation of other variants’ base reproduction numbers by multiplying it by the corresponding factor *f*. Our model also included a small random influx of variants from abroad, so new variants appear in our system by importation. This influx is thus essential to explain the sudden emergence of new variants among sequenced samples. The above implies deviations from an ideal sampling (binomial distribution). Consequently, we also incorporate a correction factor that penalises non-ideal measurements with more significant errors than expected under binomial sampling (see “Methods”).

We used two data sources to infer the variants’ relative transmissibility and other parameters in our model: (1) the weekly averaged variant share (i.e., the fraction a given variant represents of the total samples) to constrain our model, assuming that these observations follow a multinomial distribution. (2) The daily number of (largely non-sequenced) observed new cases to infer the absolute prevalence of the variants in time. Our method differs from the phylodynamic inference of population growth rates as implemented in BEAST 2^56,57^ in that it does not build phylogenetic trees, but only groups the different variants together, which substantially simplifies the inference when data is scarce.

To characterise the genetic diversity of SARS-CoV-2 variants circulated in Chile, we selected 2650 complete sequences of SARS-CoV-2 (over 29,000 base pairs) and built a phylogenetic tree. Then, we compared those sequences selected to SARS-CoV-2 reference sequences, assigned them to clades, and determined their position within the reference phylogenetic tree using the Nextclade Web tool^58^. We use the normalised Total Mutational Load (nTML)^59^ as a proxy for mutation enrichment of different parts of the genome (see “Methods”).

## Results

### Transmissibility of most predominant SARS-CoV-2 variants in Chile

**Figure 1:**
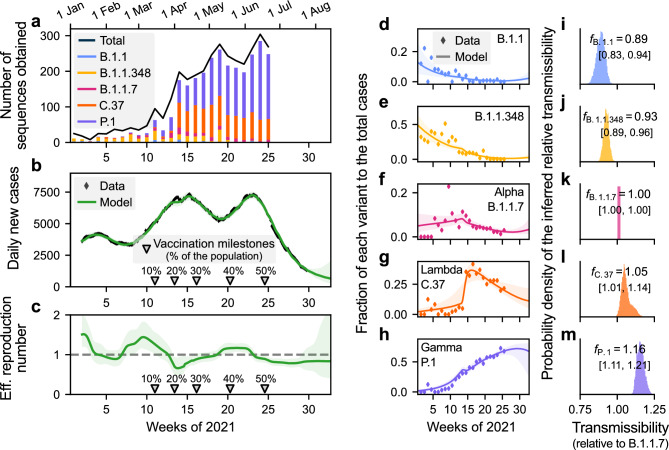
Bayesian inference enables individual assessment of the contribution of different SARS-CoV-2 variants to the spread of COVID-19. (**a**) Throughout 2021, five SARS-CoV-2 variants were identified as predominant in Chile, two considered Variants of Concern (VoC) by the WHO (Alpha, and Gamma), one Variant of Interest (Lambda), and two other unflagged lineages (B.1.1 and B.1.1.348). The total black line also included other non-predominant variants. Assuming that the contribution of each variant to the spreading dynamics (**a–c**) is proportional to their share (i.e., the fraction they represent of the total samples, **d–h**), we quantified their transmissibility compared to the Alpha variant (**i–m**). The Lambda and Gamma variants showed a 1.05 (95% CI [1.01,1.14]) and 1.16 (95% CI [1.11,1.21]) fold higher reproduction number than the Alpha variant. Other variants had a comparatively lower influence on the spread. Shaded areas in the **b–h** panels account for the 95% credible intervals of the model fit. Complementary parameters and variables are summarised in Supplementary Fig. S1.

Among the samples collected between January and June 2021 (n=3443) we identified 86 different SARS-CoV-2 lineages. However, after filtering the dataset to consider only those that represent at least 20% of the total samples during one weekly observation period (n=2920) we identify five predominant variants: Gamma (labelled as Variant of Concern, VoC), Lambda (labelled as Variant of Interest, VoI), Alpha (VoC), B.1.1.348, and B.1.1 (see Fig. 1a). The Gamma VoC, first reported in November 2020 in Manaus, Brazil^60^, was the dominant variant in Chile from May 2021 on, counting 1614 samples in the period analysed. It was followed by the Lambda VoI, with 838 samples. The Alpha VoC, to date reported in 183 countries around the world^61^, was detected only 158 times in Chile. In addition to those VoCs and VoI mentioned before, we have identified 252 samples classified as B.1.1.348 and 58 as B.1.1.

The Bayesian model fitted the daily number of cases well (Fig. 1b) by adapting the effective reproduction number (Fig. 1c) and also modelling the share of the different variants over time (Fig. 1d–h). The emergence and sudden increase in the predominance of the Lambda variant around week 12 (cf. Fig. 1g) is unlikely to be due solely to community transmission. As Lambda cases were zero or extremely low, this increase can be explained by an abrupt influx of cases (Supplementary Fig. S2d), which acted as a seed for community transmission.

We found that the inferred relative reproduction number was the lowest for the non-VoC variants B.1.1 and B.1.1.348 (Fig. 1i,j). From all the variants of concern and interest, our reference variant Alpha had the lowest transmissibility (Fig. 1k–m), followed by Lambda ($f_\mathrm{Lambda}=1.05$, 95% CI: [1.01, 1.14]) and Gamma with the highest reproduction number ($f_\mathrm{Gamma}=1.16$ (95% CI: [1.11,1.21]).

### Mutational load of the Spike gene correlates with variant transmissibility

**Figure 2:**
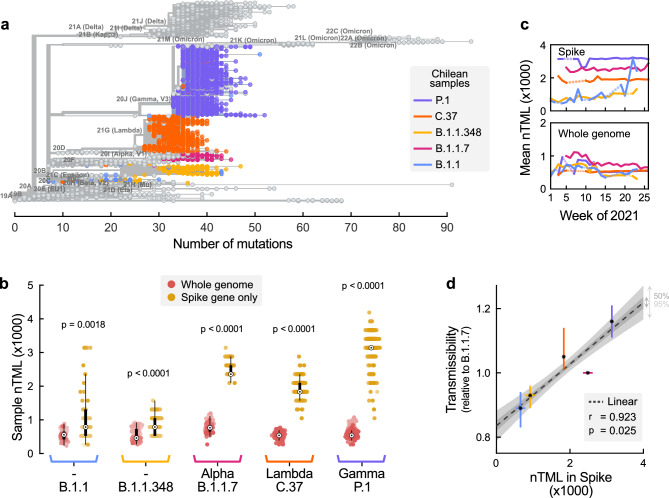
Predominant variants are enriched with mutations in the Spike gene. (**a**) The Nextclade-based (https://clades.nextstrain.org/tree) phylogenetic tree of the SARS-CoV-2 variants isolated in Chile, visualised using Auspice online tool (https://auspice.us/ ) based on n = 2650 SARS-CoV-2 cases. The sequences are placed on a global reference tree (grey brunches and nodes), and clades are assigned to the nearest neighbour, while the branches with coloured circles represent lineages from Chile. (**b**) The normalised Total Mutational Load (nTML) indicates that the Spike gene is enriched in mutations compared to the entire genome for all analysed variants. The apparent discreteness of the Spike nTML traces is due to the shorter gene length. The white points denote the median, black boxes denote the interquartile ranges, and whiskers (thin black lines) extend until at most 1.5 times the length of the interquartile range, and dot opacity denotes the time when samples were collected (light $\rightarrow$ old, dark $\rightarrow$ recent). Significance levels were determined with an u-test, see Supplementary Table S2). **c.** The most predominant variants do not show a considerable drift in their average nTML over time. Dotted lines account for weeks when the variants were not observed. **d.** There is a marked and significant positive correlation between nTML in Spike and the variants’ relative transmissibility (median r = 0.923, p-value = 0.025). Vertical error bars are those reported in Figure 1, asterisks denote median values, and horizontal error bars were estimated through bootstrapping.

Although SARS-CoV-2 variants share an evolutionary history, we observe a broad dispersion in the number of mutations (i.e., TML) even within lineages. On the other hand, while some samples of different lineages seem to have the same absolute number of mutations (seen, e.g., when drawing vertical cuts in Fig. 2a), they have different mutational profiles and, therefore, are classified in different clades according to the PANGOLIN criteria. These differences, which are indistinguishable when analysing the total number of mutations, might become evident when studying the relative enrichment in mutations of different regions of the genome (i.e., the nTML).

We computed the nTML for both the whole genome and solely for the Spike gene for Chile’s predominant circulating variants (cf. to Fig. 2b), using as the reference the SARS-CoV-2 Wuhan-Hu-1 isolate (Accession: NC_045512.2). Observed mutations were typically missense, i.e., cause an observable change in the generated amino acid sequence, and have been reported to also impact the function of certain translated proteins in SARS-CoV-2^62^. We partially eliminate the codependency between nTML in Spike and the whole genome by subtracting the number of mutations in Spike from those in the whole genome. We observed a statistically significant enrichment in mutations in the Spike gene in all lineages. Among them, the Gamma VoC had the highest number of mutations in the Spike gene, followed by Alpha, Lambda, B.1.1.348, and finally B.1.1 with the lowest nTML (Fig. 2b). The Spike gene showed a marked dispersion in the nTML in all samples compared to the whole genome. There were no relevant temporal variations in nTML except for lineage B.1.1, where the mean nTML in Spike seems to increase at the end of the analysed period (Fig. 2c). However, this observation can be an artefact induced by the low number of samples found for this lineage.

The main difference between variants was the degree of mutation enrichment in the Spike gene, quantified by their median nTML. Furthermore, we found a marked linear correlation between the nTML in Spike and the relative transmissibility of the most predominant lineages (piece-wise linear correlation $r= 0.923$, p-value=0.025,  Fig. 2d).

### Local samples differ from reference genomes

Chilean samples of different variants systematically exhibit mutation patterns not present in the reference lineages, i.e., they drift from the minimal list of defining mutations presented in Supplementary Table S1. We studied the mutational profile of Chilean samples categorised among the five lineages studied here and calculated the frequency of non-defining mutations. Table 1 summarises the results after filtering for the mutations present in at least 25% of the samples detected in the time frame analysed.

The mutations found in Chilean samples are not limited to the Spike gene or structural domains. All variants share the P322L mutation in the RdRp complex (NSP12), which is responsible for replicating and transcribing the virus genome^63^. Closely related lineages tend to accumulate similar non-defining mutational patterns, such as mutations F106F and L357F in NSP3, R203R (N), and V149F in (NSP6), which were frequently observed among both B.1.1 and B.1.1.348 samples. B.1.1 samples also accumulated the A262S and G1167A mutations in the Spike protein, which have been reported to significantly increase the virus’s infectivity and have a synergistic effect when occurring simultaneously^64^.

On the other hand, lineages start accumulating mutations that become defining for the more evolved variant. For example, the NSP6_S106 mutation, detected among all lineages except for B.1.1.348, and especially prevalent for the VOI/VOC, became defining for the Omicron variant^65^. Mutations N_S2Y and ORF3a_G174D were observed in a large fraction of the B.1.1 samples, and they became defining for the more evolved B.1.1.348 lineage. The R346G mutation in the Spike protein, detected in B.1.1.348 samples, became defining for the Mu VoI^66^. Other less prevalent examples are Spike_N501Y and Spike_H655Y, respectively, from lineages B.1.1 and B.1.1.348 to Gamma (see Supplementary data for details).

**Table 1:** Most predominant non-defining mutations in Chilean samples.

B.1.1		B.1.1.348		B.1.1.7		C.37		P.1	
Mutation	Frequency	Mutation	Frequency	Mutation	Frequency	Mutation	Frequency	Mutation	Frequency
NSP12_P322L	98,3%	NSP12_P322L	100,0%	NSP6_S106	100,0%	NSP12_P322L	100,0%	NSP3_K977Q	100,0%
NSP3_F106F	48,3%	NSP6_V149F	100,0%	Spike_H69	100,0%	NSP6_S106	99,9%	NSP12_P322L	99,9%
N_R203R	48,3%	NSP3_L357F	96,8%	NSP3_A890D	99,4%	NSP3_F1569V	99,6%	NSP6_S106	99,9%
NSP3_S1717L	34,5%	NSP12_N733N	44,8%	N_D3E	99,4%	NSP3_P1469S	99,4%	NSP3_S370L	99,5%
NSP4_L447F	34,5%	NSP13_L438L	44,8%	N_D3H	99,4%	Spike_T859N	99,4%	N_S202C	98,9%
NSP6_V120I	34,5%	NSP4_A416A	44,8%	N_D3V	99,4%	NSP4_T492I	99,3%	N_S202T	98,9%
NSP6_V149F	27,6%	ORF3a_F43F	44,8%	NSP12_P322L	98,7%	NSP4_L438P	99,0%	NSP13_E341D	98,3%
Spike_G1167A	27,6%	NSP3_F106F	44,4%	NSP3_T183I	98,1%	NSP3_T428I	98,5%		
NSP3_L357F	25,9%	N_R203R	43,7%	NSP3_I1412T	96,8%	NSP5_G15S	86,1%		
N_S2Y	25,9%	Spike_R346K	40,9%	NSP13_K460R	43,3%				
ORF3a_G174D	25,9%			NSP14_E347G	42,0%				
Spike_A262S	25,9%			NSP3_I1460V	42,0%				
				ORF6_T21I	42,0%				
				ORF8_K68*	32,5%				

### Temporal drift of variants’ mutational signatures

**Figure 3:**
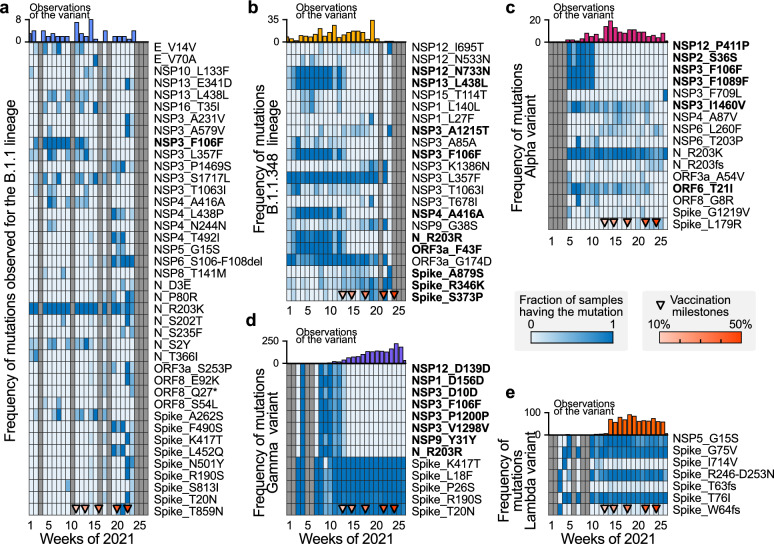
Signatures of the settlement, replacement, and selection of mutations in the different observed lineages of SARS-CoV-2. Throughout 2021, the set of mutations that are present in the analysed samples of the predominant lineages has changed. This temporal evolution of the mutational footprint of the lineages can be quantified by the proportion of the analysed samples which present a given mutation. We selected mutations with the largest temporal variability for each lineage, and we present their evolution as a heat map. (**a–e**) Evolution of the fraction of the samples presenting a given mutation for the B.1.1 (**a**), B.1.1.348 (**b**), Alpha (**c**), Gamma (**d**), and Lambda (**e**) variants, respectively, with their number of observations. Triangle markers at the lower end of each heat map account for the progress in vaccination.

We analysed the temporal trends for the frequency of non-defining mutations observed among the samples for each lineage, selecting only those mutations that present the most considerable variance. Samples belonging to B.1.1 and B.1.1.348 lineages (Fig. 3a,b) presented the highest variability in their mutational profile. However, their occurrence was less frequent than the other lineages (especially for B.1.1), thus the subsampling-induced noise can explain part of the variability.

Lineages with the highest relative reproduction number and nTML, i.e., Gamma, Lambda, and Alpha (cf. to Fig. 2), persisted throughout vaccine roll-out as samples collected in this period were still identified as such. However, we observed specific changes in these variants’ mutational signatures (and frequencies) over time. In particular, we can observe that the frequency of some mutations in the analysed samples drastically reduced as vaccination progressed. For example, the P411P (NSP12 gene), S36S (NSP2 gene), F106F and F1089F (NSP3 gene) mutations in Alpha (Fig. 3c) and the D156D (NSP1 gene), Y31Y (NSP9 gene), D139D (NSP12 gene), R203R (N gene) and D10D, P1200P, V1298V (NSP3 gene) in Gamma variants (Fig. 3d) were not frequently observed after reaching the $\approx 20\%$ fully vaccinated population milestone. On the other hand, mutations in the B.1.1 (Fig. 3a) variant are highly variable, which could be a subsampling artefact, and the mutational profile of the Lambda variant does not significantly change over time or with the progress of vaccination (Fig. 3e).

## Discussion

In this work, we quantified the relative transmissibility of Chile’s predominant SARS-CoV-2 variants using a Bayesian model for disease spread tailored for conditions with limited genomic surveillance. The time frame of our analysis is limited to January and June 2021, as the representativeness of the sampling protocol was compromised after the importation of the Delta VoC as samples suspected to be Delta were prioritised for sequencing^11,15^. We estimate the effective reproduction number ($R_{\text {eff}, t}$) and thereby the relative transmissibility of all variants relative to the Alpha VoC. Due to the *effective* nature of the reproduction number inferred, an increase in variant-specific reproduction numbers is not necessarily an increase in the variant’s base transmissibility but could reflect the particularities of the population where it spreads, e.g., overall immunity levels.

We found that the relative transmissibility of the Gamma VoC compared to Alpha was $f_\mathrm{Gamma}=1.16$ (95% CI: [1.11, 1.21]), which is in good agreement with the literature. For example, a contemporary study using pooled estimates across several countries reports $f_\mathrm{Gamma}=1.1$ (95% CI: [1.03, 1.17])^67^. Another study reports $f_\mathrm{Gamma}=1.09$ (95% CI: [0.82, 1.44]) and $f_\mathrm{Gamma}=1.09$ (95% CI: [0.96, 1.25]) using respectively 2.1 and 6.4 million of sequences worldwide^10,68^. (Note that the results in^10,68^ are relative transmissibility and 95% confidence intervals with respect to the wild type (WT) lineage ($R/R_{WT}$ 95% CI [lb,ub]), so the relative transmissibility between variants and their 95% CI were estimated as R1/R2 [lb1/ub2, ub1/lb2]). Our estimates reflect better the situation observed in Manaus, Brazil, for P.1, with peak transmissibility at reproduction numbers close to 5^60^.

Our findings for the relative transmissibility of the Lambda VoI, $f_\mathrm{Lambda}=1.05$ (95% CI: [1.01, 1.14]), are also within the ranges reported in^10,68^, where it was estimated as $f_\mathrm{Lambda}=1.05$ (95% CI: [0.8, 1.39]) and $f_\mathrm{Lambda}=1.12$ (95% CI: [1.03, 1.21]) using respectively 2.1 and 6.4 million of sequences worldwide^10,68^. Although in Perú, a highly affected neighbour country, the transmissibility of Lambda was not quantified, it has been shown that it replaced other circulating variants swiftly^69–71^, thus arguing in favour of a higher value of $f_\mathrm{Lambda}$. However, in Chile, the Lambda variant did not fully replace the Gamma VoC in the timeframe analysed, being consistent with our finding that $f_\mathrm{Lambda}<f_\mathrm{Gamma}$ in these regional settings. As time evolved, the partial immune escape of Lambda helped replace Gamma in some regions and thus yielded a higher $f_\mathrm{Lambda}$, as reported in the latest version of^10^.

The mutational characterisation presented in this work provides valuable preliminary insights into the genomic factors associated with the transmissibility of SARS-CoV-2 variants. In particular, we found a statistically significant enrichment of mutations in the Spike gene compared to the rest of the genome. Moreover, this enrichment in the Spike protein exhibited a strong positive correlation with the transmissibility of the analysed variants. This enrichment can be attributed to the critical role of the Spike protein, which facilitates the virus’s entry into host cells^72^. Interestingly, we found that the Lambda variant has a lower nTML in the Spike gene than the Alpha variant, yet it exhibits higher relative transmissibility. This may be due to the presence of the L452Q and F490S mutations, which have been identified as critical drivers of Lambda’s spread in South America^73^.

Specific mutations in the Spike gene of Gamma and Lambda variants were crucial for the survival of these variants during vaccine roll-out. For the Gamma variant, Spike mutations have been associated with enhanced transmissibility (N501Y) and with partial immune escape (K417T and E484K)^74^. For the Lambda variant, Spike mutations L452Q, F490S and deletion 246-252 conferred partial immune escape against neutralising antibodies elicited by CoronaVac and a higher infectiousness than the Gamma variant^75^. Although all vaccines, and therefore vaccine-elicited antibodies, are targeted towards the SARS-CoV-2 Spike protein, mutational data suggests that evolutionary pressure was also exerted on other viral genes. This fact becomes evident after reaching the 20% vaccination milestone, i.e., the eldest 20% of the population was vaccinated on weeks 13–14. Many earlier mutations in non-Spike genes disappeared during this transition, while others increased their frequency (cf. Supplementary Table S5 and Fig. 3). On the one hand, receding lineages (B.1.1 and B.1.1.348) tended to develop new Spike mutations before disappearing. For example, the S373P mutation in the RBD domain has been reported to partially escape immunity granted by mRNA vaccines and decrease plasma therapy success^76^, while R346K conferred higher transmissibility^77^. On the other hand, thriving lineages (Gamma and Lambda) tended to conserve and fix pre-existing Spike mutations. All lineages, except for Lambda, consistently developed non-Spike mutations during vaccine roll-out. The remaining variants were probably selected through epistatic fitness of a restricted protein subgroup, particularly Spike (S) and the nucleocapsid (N) protein^78^. However, we cannot infer a causal relationship between extinction and the appearance of mutations with the vaccination process with the data we have: whether there is causality behind this correlation should be separately studied.

Our modelling approach enables quantifying the relative transmissibility of different variants spreading simultaneously in settings with limited GS. Overcoming subsampling, besides requiring modelling the possibility of imperfect sampling, comes at the cost of simplifying assumptions. We assume that the generation interval does not vary between variants, which is a common assumption in the field (used, e.g., in^10^). It is known that ignoring potential differences in the generation interval of SARS-CoV-2 variants might affect the estimate of their relative transmissibility^79^. However, these are minimal when $R_\mathrm{eff}\approx 1$. Further, serial intervals are not drastically different between these VoCs (which is not necessarily the case for Delta and Omicron VoCs,^80^), and the credible intervals in their estimations overlap^81^—further justifying our choice of timeframe of analysis. Besides, we assumed that the influx of infections (and thereby, of variants) was proportional to the COVID-19 incidence in neighbouring countries and evenly distributed across all tracked variants. Currently, the influx corresponds to a tiny percentage of the total cases ($\le {5}\%$, cf. Fig. 1b and Fig. S2a–e). However, more exact modelling would be required when neighbouring countries have considerably more cases than the country of study, as the influx can considerably affect community spread. Unfortunately, GS in neighbouring countries was not representative enough to allow us to incorporate it into our workflow (see, e.g.,^24^). More details regarding model robustness are presented in the Supplementary Materials.

Quantifying the transmissibility of emerging public health threats and understanding the mechanisms behind them is crucial for guiding effective control and prevention strategies for emerging threats and diseases with nontrivial endemic patterns^82,83^. The methodology proposed in this work can promptly quantify the relative transmissibility of viral variants even in situations with limited GS. Besides providing timely insights on the regional characteristics of the spread of Gamma, Lambda and other SARS-CoV-2 variants, our study provides a tool for countries with limited capacity for GS to maximise the information they extract. With this methodology and ensuring a representative sampling (following, e.g.,^11^), we demonstrate the benefits of running GS programmes and their crucial role in public health.

## Methods

### Nucleic acid extraction and amplification

Nasopharyngeal samples, previously confirmed as positive for SARS-CoV-2, were used for total nucleic acid extraction using the automated system Zybio EXM 6000. Reverse transcription for cDNA synthesis was performed with SuperScript III One-Step RT-qPCR System with Platinum Taq Kit, RNase OUT (Invitrogen) with 2 mM random primers and 4.5 $\mu$M DTT at 55$^{o}$C for 60 min. cDNA was amplified based on COVID-19 ARTIC Illumina Library Construction and Sequencing Protocol V.3 (Farr, 2020), generating two pools with 400 pb length amplicons covering the whole viral genome.

### Library preparation

DNA fragments from each pool were mixed together and the library was prepared with Illumina DNA PREP kit (Illumina, San Diego, CA, USA), purified using Agencourt AMPure XP beads (Beckman Coulter, Brea, CA, USA) and quantified by Victor Nivo Fluorimeter (Perkin Elmer) using Quant-it dsDNA HS Assay Kit (Invitrogen). DNA libraries were sequenced in a MiSeq (Illumina) using a 300 cycles kit. Around 0.3 GB of data was obtained for each sample.

### Whole Genome Sequence analysis

Sequence quality was analysed with FastQC software v0.11.8. Readings were filtered and trimmed with BBDuk software considering a minimum of 36 bases length and quality above>Q20. Genome assembly was performed with IRMA software v0.9.3^45^ using as the reference sequence the NCBI entry NC_045512.2. We aligned the genomes with MAFFT v7.458 and filtered them by a genome coverage of >95% and a mean depth of 1000x. The lineages for the assembled sequences were assigned with Pangolin v3.1.5^47^. Final genomes with epidemiological metadata were submitted to https://www.gisaid.org/ for the final quality check and the corrected lineages. We analysed 3956 SARS-CoV-2 sequencing samples in the Chilean Public Health Institute (ISP) obtained from January to June of 2021, of which 3443 obtained good quality and genome coverage results We used Pangolin to assign the variant classification for samples with good quality measures.

### Determination of normalised Total Mutational load

From the mutational data, we implemented an $m \times n$ mutation count matrix by considering all types of mutations and deletions. In the matrix, *m* is the number of samples (2726, considering only those belonging to the five lineages studied herein), and *n* is the number of genes (25 genes). Therefore, the value in entry $V_{i,j}$ indicates the number of mutations and deletions of gene *j* in the sample *i*. Later, we computed the normalised Total Mutational Load (nTML), equivalent to the total number of mutations, divided by the length of the reference of the Spike gene and the whole genome by 1 kb (kilobases) for each sample

1 $$\begin{aligned} \text {nTML}_{i}={ \frac{1000}{w_i} }\cdot \sum _{j=0}^{j=m}V_{i,j}, \end{aligned}$$

where $w_i$ accounts for the sequence length, 3821 and 29903 Kbp for the Spike gene and whole genome, respectively. We then studied whether there was a statistically significant enrichment of mutations in the Spike gene. For that, we first applied a Levene’s test to evaluate whether, for a given lineage, the distributions of nTML for the whole genome and the Spike gene only have equal or different variances. Then, as the test confirmed that variances were different for all lineages, we used a non-parametric Mann-Whitney *U* test to assess whether the medians of the categories were significantly different for every variant. Results for both assessments are summarised in Supplementary Table S2.

### Correlation between nTML and relative transmissibility

**Figure 4:**
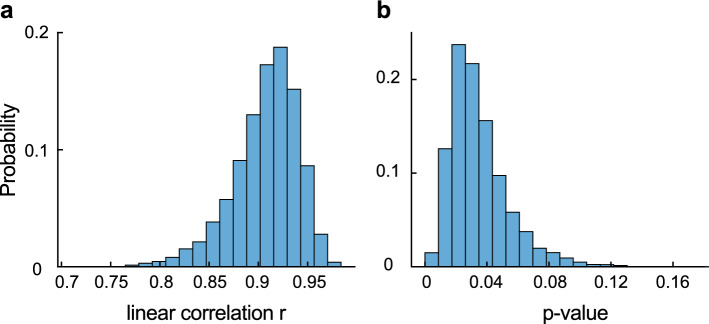
Robustness check: linear correlation between nTML in spike and variant transmissibility. Probability-normalised histograms for the linear correlation coefficient (**a**) and the associated p-value (**b**) in the Monte Carlo-inspired experiment to test for robustness. We see that the correlation is statistically significant for most of the hypothetical curves.

In order to explore whether the correlation between nTML and relative variant transmissibility is statistically significant, we proceeded as follows. First, we determine the median and 95% confidence intervals for the variant transmissibility *f* (result from the inference process) and the nTML in spike (using bootstrapping). We estimate the standard deviation of the variables assuming a normal distribution (i.e., $\sigma \approx 1/4~CI$). We design a Monte Carlo-inspired experiment, where at each step, we draw pairs $(nTML_i,f_i)$ for each variant, perform a linear regression, and calculate the piece-wise linear correlation coefficient and its associated p-value. We repeat the experiment 10000 times. We delimit confidence bands associated with the 50% and 95% of the linear regressions and analyse the distribution of correlation coefficients and p values. We find that for all hypothetical realisations of the experiment, the correlation coefficient is high, and the vast majority of them are statistically significant, showing that our results are robust (cf. to Fig 4a, b, for the stats distributions).

### Inferring the variant-specific contribution to the spread

We built our model on top of our existing spreading dynamic model^54^ to assess the relative transmissibility of the different variants in Chile. Given additional data, this model can be easily adapted for other countries or time frames.

We simulated the spread of each variant independently whereby the susceptible pool *S* was shared across the different variants. For each variant *v* we computed the number of newly exposed $E_{v}$ iteratively given a prior distribution $E_{v,0}$ and the generation interval distribution *g* with hyperprior *m*. This follows the work of^50–52^. To account for non-pharmaceutical interventions or other measures against the spread we introduced the time-dependent effective reproduction number $R_{\text {base},t}$, which is allowed a change every 14 days relative to the previous reproduction number.

For each variant *v* the effective reproduction number was modulated by the time-invariant factor $f_v$, called relative reproduction number in the text, such that the effective reproduction number of variant *v* is $R_{\text {eff}, v, t} = f_v \cdot R_{\text {base},t} \frac{S_t}{N}$. We fixed $f_v = 1$ for the Alpha variant. Additionally, to account for cases induced by travel we also add a small random influx $\Phi _v$ for each variant *v* which was scaled by the reported case numbers in the neighbouring countries $M_t$ (we used Argentina, Peru and Brazil). In discrete form the spreading dynamics in our model read as:

2 $$\begin{aligned} E_{v,t}&= \frac{S_t}{N} f_v R_{\text {base}, t} \sum _{\tau =0}^{10} E_{v,t-1-\tau } g_\tau + \Phi _{v,t} M_t, \end{aligned}$$

3 $$\begin{aligned} S_{t}&= S_{t-1} - \sum _{v} E_{v,t-1}, \end{aligned}$$

4 $$\begin{aligned} g_\tau&= \text {LogNormal}(\tau ;\mu =m,\sigma =0.4), \end{aligned}$$

5 $$\begin{aligned} m&\sim \text {Normal}(\mu =4,\sigma =1). \end{aligned}$$

Where *N* is the population size of our considered country (Chile). The prior is a little longer than the estimates of the generation interval of the Delta variant^84,85^, but shorter than the estimated serial interval of the original strain. The susceptible pool gets initialised with the population size. The prior distributions for the initial new cases of each variant $E_{v,0}$ are essentially a flat prior (as described in ^54^).

6 $$\begin{aligned} E_{v,0}&\sim \text {HalfCauchy}(\sigma =100) \quad \forall v, \end{aligned}$$

The time-invariant contribution factors $f_v$ were set to the same value for each variant to incorporate no prior knowledge about a specific variant’s contribution. Further, we choose a median of one as this is used as a multiplicative factor, this prior can be seen as relatively uninformative.

7 $$\begin{aligned} f_v&\sim \text {LogNormal}(\mu = 0, \sigma = 1) \quad \text {for } v \in \{ \text {B.1.1, B.1.1.348, Gamma, Lambda} \}, \end{aligned}$$

8 $$\begin{aligned} f_{\text {Alpha}}&= 1, \end{aligned}$$

9 $$\begin{aligned} f_{\text {others}}&= f_{\text {others}}(t) \end{aligned}$$

In addition to the five variants mentioned in the main text, we also include in our model the share of sequenced cases not categorised into these five variants ($f_{\text {others}}$). In contrary to the other five main variants, the relative reproduction number of these other variants is allowed to vary over time (described later). The external input $\Phi _{v,t_w}$ was modelled in a weekly fashion, indexed by $t_w$, to decrease the number of variables to be estimated. Here we choose a small contribution for each variant as we expect influx to be less predominant than in-country infections, we assume 0.0005 %.

10 $$\begin{aligned} \Phi _{v,t_w}&\sim \text {HalfStudentT}_{\nu =4}(\sigma =0.0005) \quad \forall v, \forall t_w. \end{aligned}$$

Let $y_{v,t}$ be the measured number of samples successfully sequenced (from samples having a positive RT-qPCR test), corresponding to variant *v*. Let $n_t$ be the total number of sequenced samples and $\tau _{v,t}$ the inferred relative case numbers of the variant *v* at time *t* compared to the total non-variant case numbers. If we model the number of samples $y_{v,t}$ corresponding to a variant *v* as a multinomial random variable, and assuming that samples collected for sequencing are independent, we can build the multinomial likelihood function for our model with our real-world data *y* and *n* and the fraction of variant $\tau$ from the model:

11 $$\begin{aligned} y_{v,t}&\sim \text {Multinomial}(p_v = \tau _{v,t}, n=n_t) \quad \forall t. \end{aligned}$$

The fraction $\tau _{l,t}$ is obtained from the model by the fraction between daily cases of a variant *v* and total daily cases.

12 $$\begin{aligned} \tau _{v,t} = \frac{E_{v,t}}{\sum _{v} E_{v,t}} \end{aligned}$$

However in our model, we do not use this multinomial likelihood function but instead parameterise our model using the conjugate distribution, the Dirichlet distribution. In theory, it is equivalent to using the multinomial distribution. The advantage is that we can add a factor *w* that parameterises an eventual non-optimal sampling strategy, for example, samples that are not being perfectly randomised across the country but are correlated to some extent. This has mathematically the consequence that the measured fractions $y_{n,t}/n_t$ are all reduced by a factor *w*. Thus, the resulting likelihood function is given by:

13 $$\begin{aligned} \tau _{v,t}&\sim \text {Dirichlet}\left( \alpha =w \cdot \frac{y_{v,t}}{n_t} + 1 \right) \quad \text {with} \end{aligned}$$

14 $$\begin{aligned} w&\sim \text {Gamma}(\alpha =5,\beta =5) \end{aligned}$$

To infer the slowly changing reproduction number we introduce sigmoidal change points relative to the previous reproduction number whereby the priors for the date of occurrence *d* of the change point *c* are set every 14 days. The transient length *l* such as the date *d* of each change point *c* is defined relatively flat to express our uncertainty in these values.

15 $$\begin{aligned} R_{\text {base}, t}&= \exp \left( \sum _c \gamma _{c}(t)\right) \end{aligned}$$

16 $$\begin{aligned} \gamma _{c}(t)&= \frac{\Gamma _c}{1+e^{-4/l_{c} \cdot (t - d_{c})}} \end{aligned}$$

17 $$\begin{aligned} d_c&\sim \text {Normal}(\mu =14, \sigma =5)&\forall c \end{aligned}$$

18 $$\begin{aligned} l_c&\sim \text {Normal}(\mu =20,\sigma =6)&\forall c \end{aligned}$$

19 $$\begin{aligned} \Gamma _c&\sim \text {Normal}(\mu =0,\sigma =0.2) + \Gamma _{c-1}&\forall c\ne 0 \end{aligned}$$

20 $$\begin{aligned} \Gamma _0&\sim \text {Normal}(\mu =1,\sigma =0.2) \end{aligned}$$

For the five variants that we focused on in the main text, $R_{\text {base}, t}$ is multiplied by a time-invariant relative reproduction number $f_v$. For the spread of the ‘other variants’ that we modelled separately, we multiplied this $R_{\text {base}, t}$ by a time-dependent $f_{\text {others}}(t)$ as the mixture of variants can slowly change over time. We assumed the this change is slower than the $R_{\text {base}, t}$:

21 $$\begin{aligned} f_{\text {others}}(t)&= \exp \left( \sum _c \gamma _{\text {others},c}(t)\right) \end{aligned}$$

22 $$\begin{aligned} \gamma _{\text {others},c}(t)&= \frac{\Gamma _{\text {others}, c}}{1+e^{-4/l_{\text {others},c} \cdot (t - d_{\text {others},c})}} \end{aligned}$$

23 $$\begin{aligned} d_{\text {others},c}&\sim \text {Normal}(\mu =14, \sigma =5)&\forall c \end{aligned}$$

24 $$\begin{aligned} l_{\text {others},c}&\sim \text {Normal}(\mu =20,\sigma =6)&\forall c \end{aligned}$$

25 $$\begin{aligned} \Gamma _{\text {others},c}&\sim \text {Normal}(\mu =0,\sigma =0.2) + \Gamma _{\text {others},c-1}&\forall c\ne 0 \end{aligned}$$

26 $$\begin{aligned} \Gamma _{\text {others},0}&\sim \text {Normal}(\mu =1,\sigma =0.2) \end{aligned}$$

In addition to the sequenced samples, we constrain our model using the publicly reported case numbers (in Chile) $C_t$ aggregated by the Johns Hopkins University ^86^. We sum over the newly infected pools for all variants to obtain the total number of new infections $E_t = \sum _v E_{v,t}$. These are then delayed with the LogNormal kernel with mean delay *D* to account for a reporting delay and further modulated by a weekly absolute sinus function parameterised by an amplitude $h_w$ and an offset $\chi _w$.

27 $$\begin{aligned} \hat{C_t}&= (1-h(t)) \cdot \sum _{\tau =1}^{T} E_{t-\tau } \cdot \text {LogNormal}(\tau ; \mu = D, \sigma = 0.3) \end{aligned}$$

28 $$\begin{aligned}&= (1-h(t)) \cdot \sum _{\tau =1}^{T} E_{t-\tau } \frac{1}{0.3\cdot \tau \sqrt{2\pi }}e^{-\frac{\left( \log (\tau )-\log (D)\right) ^2}{2\cdot 0.3^2}} \end{aligned}$$

29 $$\begin{aligned} D&\sim \text {LogNormal}(\mu = 10, \sigma = 0.2) \quad \text { and with} \end{aligned}$$

30 $$\begin{aligned} h(t)&= (1-h_w) \cdot \left( 1 - \left| \sin \left( \frac{\pi }{7} t- \frac{1}{2}\chi _w\right) \right| \right) \end{aligned}$$

The likelihood given the reported case numbers $C_t$ is then modelled by a StudentT distribution and quantifies the similarity between the model outcome and the available real-world time series. The scale factor $\kappa$ heuristically incorporates the measurement noise.

31 $$\begin{aligned} C_t&\sim \text {StudentT}_{\nu = 4}\left( \mu = \hat{C_t}, \sigma = \kappa \sqrt{\hat{C_t}}\right) \quad \text {with} \end{aligned}$$

32 $$\begin{aligned} \kappa&\sim \text {HalfCauchy}(\sigma =10) \end{aligned}$$

For a complete list of model parameters and priors see Supplementary Tables S3 and S4 respectively.

To estimate the parameters of the Bayesian model, we use Monte Carlo sampling. In this way, we also obtain credible intervals of the parameters and not only the maximal likelihood estimate. Specifically, the sampling was performed using PyMC3 ^87^. We use a NUTS sampler^88^, which is a Hamiltonian Monte-Carlo sampler. The chains are initialised randomly. We run 16 chains for 1200 tuning steps and sample for 1500 steps The maximum tree depth is set to 10 and the target acceptance ratio to 0.95.

To quantify whether the chain mixes well and the model is converging, we plot the values of the inferred relative transmissibility over time (Supplementary Fig. S3). All variants display a good mixing except Gamma (P.1). It shows a slightly bimodal behaviour. Therefore median and credible interval for the relative transmissibility of Gamma might be slightly biased, as we do not have the mathematical assurance that our model has converged for this variable.

### Supplementary Information

Supplementary Information 1.

Supplementary Information 2.

## Data Availability

Some source code for data generation and analysis is available online on GitHub https://github.com/Priesemann-Group/covid19_variants_chile. Sample sequencing was conducted at the Chilean Public Health Institute (ISP). All genomes sequenced by ISP are hosted in the GISAID Initiative^12^. Additionally, for the Bayesian inference, we used the daily case reports for Chile, Brazil, Argentina and Peru aggregated by the Johns Hopkins University^86^.

## References

[1] Plante JA, Mitchell BM, Plante KS, Debbink K, Weaver SC, Menachery VD (2021). The variant gambit: COVID’s next move. Cell Host Microbe.

[2] Van Egeren D (2020). Risk of evolutionary escape from neutralizing antibodies targeting SARS-CoV-2 spike protein. MedRxiv.

[3] Singh D, Yi SV (2021). On the origin and evolution of sars-cov-2. Exp. Mol. Med..

[4] Rogozin IB (2024). Properties and mechanisms of deletions, insertions, and substitutions in the evolutionary history of sars-cov-2. Int. J. Mol. Sci..

[5] Thompson RN, Hill EM, Julia RG (2021). Sars-cov-2 incidence and vaccine escape. Lancet Infect. Dis..

[6] Contreras S, Priesemann V (2021). Risking further COVID-19 waves despite vaccination. Lancet Infect. Dis.

[7] Lavine JS, Bjornstad ON, Antia R (2021). Immunological characteristics govern the transition of COVID-19 to endemicity. Science.

[8] Cobey S, Larremore DB, Grad YH, Lipsitch M (2021). Concerns about sars-cov-2 evolution should not hold back efforts to expand vaccination. Nat. Rev. Immunol..

[9] Chen Z (2022). Global landscape of SARS-CoV-2 genomic surveillance and data sharing. Nat. Genet..

[10] Obermeyer F (2022). Analysis of 6.4 million sars-cov-2 genomes identifies mutations associated with fitness. Science.

[11] Oróstica KY (2022). New year, new SARS-CoV-2 variant: Resolutions on genomic surveillance protocols to face omicron. Lancet Reg. Health-Am..

[12] Yuelong S, John M (2017). Gisaid: Global initiative on sharing all influenza data—from vision to reality. Eurosurveillance.

[13] Armstrong GL (2019). Pathogen genomics in public health. N. Engl. J. Med..

[14] Muellner P, Stärk KDC, Dufour S, Zadoks RN (2016). Next-generation surveillance: An epidemiologists perspective on the use of molecular information in food safety and animal health decision-making. Zoonoses Public Health.

[15] Contreras S (2023). Model-based assessment of sampling protocols for infectious disease genomic surveillance. Chaos Soliton. Fract..

[16] Struelens MJ (2024). Real-time genomic surveillance for enhanced control of infectious diseases and antimicrobial resistance. Front. Sci..

[17] Re3data.Org. GISAID. re3data.org—Registry of Research Data Repositories (2022). 10.1371/journal.pone.0078080 PMC3817176 24223762

[18] Cyranoski D (2021). Alarming COVID variants show vital role of genomic surveillance. Nature.

[19] Malick MSS, Fernandes H (2021). The genomic landscape of sars-cov-2: Surveillance of variants of concern. Adv. Mol. Pathol..

[20] Bartlow AW, Middlebrook EA, Romero AT, Fair JM (2021). How cooperative engagement programs strengthen sequencing capabilities for biosurveillance and outbreak response. Front. Public Health.

[21] Mohamed H, Mohamed A, Kareem AM (2016). Limited resources of genome sequencing in developing countries, challenges and solutions. Appl. Transl. Genom..

[22] Sachs JD (2022). The lancet commission on lessons for the future from the covid-19 pandemic. The Lancet.

[23] Onywera H (2024). Boosting pathogen genomics and bioinformatics workforce in Africa. Lancet. Infect. Dis.

[24] Brito AF (2022). Global disparities in sars-cov-2 genomic surveillance. Nat. Commun..

[25] Mena GE (2021). Socioeconomic status determines covid-19 incidence and related mortality in Santiago, Chile. Science.

[26] Gozzi N, Tizzoni M, Chinazzi M, Ferres L, Vespignani A, Perra N (2021). Estimating the effect of social inequalities on the mitigation of covid-19 across communities in santiago de chile. Nat. Commun..

[27] Bennett M (2021). All things equal? heterogeneity in policy effectiveness against covid-19 spread in chile. World Dev..

[28] Freire-Flores D (2021). On the heterogeneous spread of covid-19 in chile. Chaos Soliton. Fract..

[29] Contreras S (2020). A multi-group SEIRA model for the spread of COVID-19 among heterogeneous populations. Chaos Soliton. Fract..

[30] Castillo A (2020). Geographical distribution of genetic variants and lineages of sars-cov-2 in chile. Front. Public Health.

[31] Sanchez-Daza A, Medina-Ortiz D, Olivera-Nappa A, Contreras S (2022). COVID-19 Modeling Under Uncertainty: Statistical Data Analysis for Unveiling True Spreading Dynamics and Guiding Correct Epidemiological Management.

[32] Ayala A, Vargas C, Elorrieta F, Dintrans PV, Maddaleno M (2023). Inequity in mortality rates and potential years of life lost caused by COVID-19 in the greater Santiago. Chile. Res. Square.

[33] Shepherd A (2021). Covid-19: Chile joins top five countries in world vaccination league. BMJ.

[34] Aguilera X, Mundt AP, Araos R, Weitzel T (2021). The story behind chile’s rapid rollout of covid-19 vaccination. Travel Med. Infect. Dis..

[35] Minsal, M. Vacunas contra sars- cov-2 utilizadas en chile mantienen altos niveles de efectividad para evitar hospitalización, ingreso a uci y muerte (2021).

[36] Brault A (2024). Direct impact of covid-19 vaccination in chile: Averted cases, hospitalizations, icu admissions, and deaths. BMC Infect. Dis..

[37] Asahi K, Undurraga EA, Valdés R, Wagner R (2021). The effect of covid-19 on the economy: Evidence from an early adopter of localized lockdowns. J. Glob. Health.

[38] Contreras S (2020). Statistically-based methodology for revealing real contagion trends and correcting delay-induced errors in the assessment of COVID-19 pandemic. Chaos Soliton. Fract..

[39] Ministerio de Salud de Chile (MINSAL) Department of Epidemiology. Tech Report: National strategy for test-trace-and-isolate (COVID-19), 3–9, July, 2021 (Estrategia Nacional de Testeo, Trazabilidad y Aislamiento COVID-19, SEMANA DEL 3 - 9 DE JULIO, 2021). https://www.minsal.cl/wp-content/uploads/2021/07/Indicadores-de-Testeo-y-Trazabilidad-13072021.pdf.

[40] Jara A (2021). Effectiveness of an inactivated sars-cov-2 vaccine in chile. N. Engl. J. Med..

[41] González-Puelma J (2021). Mutation in a sars-cov-2 haplotype from sub-antarctic chile reveals new insights into the spike’s dynamics. Viruses.

[42] Acevedo M (2021). Infectivity and immune escape of the new sars-cov-2 variant of interest lambda. MedRxiv.

[43] Romero PE (2021). The emergence of sars-cov-2 variant lambda (c. 37) in South America. MedRxiv.

[44] World Health Organization *et al*. Guidance for surveillance of SARS-CoV-2 variants: interim guidance, 9 august 2021. Technical report, World Health Organization (2021).

[45] Shepard SS, Meno S, Bahl J, Wilson MM, Barnes J, Neuhaus E (2016). Viral deep sequencing needs an adaptive approach: IRMA, the iterative refinement meta-assembler. BMC Genom..

[46] Katoh K, Misawa K, Kuma K, Miyata T (2002). MAFFT: A novel method for rapid multiple sequence alignment based on fast Fourier transform. Nucleic Acids Res..

[47] Rambaut A (2020). A dynamic nomenclature proposal for SARS-CoV-2 lineages to assist genomic epidemiology. Nat. Microbiol..

[48] Scudellari M (2021). How the coronavirus infects cells-and why delta is so dangerous. Nature.

[49] Mandavilli, A. C.d.c. internal report calls delta variant as contagious as chickenpox (2021).

[50] Fraser C (2007). Estimating Individual and Household Reproduction Numbers in an Emerging Epidemic. PLoS ONE.

[51] Flaxman S (2020). Estimating the effects of non-pharmaceutical interventions on COVID-19 in Europe. Nature.

[52] Brauner JM (2020). Inferring the effectiveness of government interventions against COVID-19. Science.

[53] Dehning J, Mohr SB, Contreras S, Dönges P, Iftekhar EN, Schulz O, Bechtle P, Priesemann V (2023). Impact of the euro 2020 championship on the spread of covid-19. Nat. Commun..

[54] Dehning J (2020). Inferring change points in the spread of COVID-19 reveals the effectiveness of interventions. Science.

[55] Davies NG (2021). Estimated transmissibility and impact of SARS-CoV-2 lineage B.1.1.7 in England. Science.

[56] Volz EM, Siveroni I (2018). Bayesian phylodynamic inference with complex models. PLoS Comput. Biol..

[57] Bouckaert R (2019). BEAST 25: An advanced software platform for Bayesian evolutionary analysis. PLoS Comput. Biol..

[58] Hadfield J (2018). Nextstrain: Real-time tracking of pathogen evolution. Bioinformatics.

[59] Oróstica KY (2022). Total mutational load and clinical features as predictors of the metastatic status in lung adenocarcinoma and squamous cell carcinoma patients. J. Transl. Med..

[60] Faria NR (2021). Genomics and epidemiology of the P.1 SARS-CoV-2 lineage in Manaus, Brazil. Science.

[61] O’Toole, A. & Hill, V. GISAID. COV-lineages: B.1.1.7. https://cov-lineages.org/global_report_B.1.1.7 (2023).

[62] Teng S, Sobitan A, Rhoades R, Liu D, Tang Q (2020). Systemic effects of missense mutations on SARS-CoV-2 spike glycoprotein stability and receptor-binding affinity. Brief. Bioinform..

[63] Lombardo D (2024). Assessing Genomic Mutations in SARS-CoV-2: Potential Resistance to Antiviral Drugs in Viral Populations from Untreated COVID-19 Patients. Microorganisms.

[64] Wang Q, Ye S-B, Zhou Z-J, Li J-Y, Lv J-Z, Bodan H, Yuan S, Qiu Y, Ge X-Y (2023). Key mutations on spike protein altering ace2 receptor utilization and potentially expanding host range of emerging sars-cov-2 variants. J. Med. Virol..

[65] Bignon E, Marazzi M, Grandemange S, Monari A (2022). Autophagy and evasion of the immune system by sars-cov-2, structural features of the non-structural protein 6 from wild type and omicron viral strains interacting with a model lipid bilayer. Chem. Sci..

[66] Fratev F (2022). R346k mutation in the mu variant of sars-cov-2 alters the interactions with monoclonal antibodies from class 2: a free energy perturbation study. J. Chem. Inf. Model..

[67] Campbell F (2021). Increased transmissibility and global spread of sars-cov-2 variants of concern as at June 2021. Eurosurveillance.

[68] Obermeyer F (2021). Analysis of 21 million sars-cov-2 genomes identifies mutations associated with transmissibility. MedRxiv.

[69] Padilla-Rojas C (2021). Genomic analysis reveals a rapid spread and predominance of lambda (c. 37) sars-cov-2 lineage in peru despite circulation of variants of concern. J. Med. Virol..

[70] Quispe-Ricalde MA (2023). Evidence of natural selection and dominance of sars-cov-2 variant lambda (c. 37) over variants of concern in cusco, peru. Adv. Virol..

[71] Vargas-Herrera N (2022). Sars-cov-2 lambda and gamma variants competition in peru, a country with high seroprevalence. Lancet Reg. Health-Am..

[72] Rathnasinghe R (2022). Characterization of SARS-CoV-2 Spike mutations important for infection of mice and escape from human immune sera. Nat. Commun..

[73] Kimura I, Kosugi Y, Jiaqi W, Zahradnik J, Yamasoba D, Butlertanaka EP, Tanaka YL, Uriu K, Liu Y, Morizako N, Shirakawa K, Kazuma Y, Nomura R, Horisawa Y, Tokunaga K, Ueno T, Takaori-Kondo A, Schreiber G, Arase H, Motozono C, Saito A, Nakagawa S, Sato K (2022). The SARS-CoV-2 Lambda variant exhibits enhanced infectivity and immune resistance. Cell Rep..

[74] Harvey WT (2021). SARS-CoV-2 variants, spike mutations and immune escape. Nat. Rev. Microbiol..

[75] Acevedo ML (2021). Infectivity and immune escape of the new SARS-CoV-2 variant of interest Lambda. MedRxiv.

[76] Mohammadi E (2021). Novel and emerging mutations of SARS-CoV-2: Biomedical implications. Biomed. Pharmacother..

[77] Wang R (2020). Characterizing SARS-CoV-2 mutations in the United States. Res. Sq..

[78] Rochman ND (2021). Ongoing global and regional adaptive evolution of SARS-CoV-2. Proc. Natl. Acad. Sci..

[79] Park SW (2022). The importance of the generation interval in investigating dynamics and control of new sars-cov-2 variants. J. R. Soc. Interface.

[80] Du Z (2022). Shorter serial intervals and incubation periods in sars-cov-2 variants than the sars-cov-2 ancestral strain. J. Travel Med..

[81] Hart WS (2022). Generation time of the alpha and delta sars-cov-2 variants: An epidemiological analysis. Lancet. Infect. Dis.

[82] Contreras S, Iftekhar EN, Priesemann V (2023). From emergency response to long-term management: The many faces of the endemic state of covid-19. Lancet Reg. Health-Europe.

[83] Wagner, J. *et al.* Societal feedback induces complex and chaotic dynamics in endemic infectious diseases. arXiv:2305.15427 (2023).

[84] Pung R, Mak TM, Kucharski AJ, Lee VJ (2021). Serial intervals in SARS-CoV-2 B.1.617.2 variant cases. The Lancet.

[85] Zhang M (2021). Transmission dynamics of an outbreak of the COVID-19 delta variant B.1.617.2 - Guangdong Province, China, May-June 2021. China CDC Wkly..

[86] Dong E, Du H, Gardner L (2020). An interactive web-based dashboard to track covid-19 in real time. Lancet. Infect. Dis.

[87] Salvatier J, Wiecki TV, Fonnesbeck C (2016). Probabilistic programming in Python using PyMC3. PeerJ Comput. Sci..

[88] Hoffman, M. D. & Gelman, A. The No-U-Turn Sampler: Adaptively Setting Path Lengths in Hamiltonian Monte Carlo. arXiv:1111.4246 [cs, stat] (2011).

