# Supplementary material for: Early mutational signatures and transmissibility of SARS-CoV-2 Gamma and Lambda variants in Chile
Source: Sci Rep. 2024 Jul 11;14:16000. doi: 10.1038/s41598-024-66885-2 (PMC11237036; doi:10.1038/s41598-024-66885-2)
Supplement: Supplementary file 2 — Supplementary Information 2. [file 41598_2024_66885_MOESM2_ESM.pdf]

Supplementary Material of Early mutational signatures and transmissibility of SARS-CoV-2 Gamma and Lambda variants in Chile

Karen Y. Oróstica[1,†], **Sebastian B. Mohr**[2,3,†], **Jonas Dehning**[2,3,†], **Simon Bauer**[2], **David Medina-Ortiz**[4], **Emil N. Iftekhar**[2,3], **Karen Mujica**[5], **Paulo C. Covarrubias**[5], **Soledad Ulloa**[5], **Andrés E. Castillo**[5], **Anamaría Daza-Sánchez**[6], **Ricardo A. Verdugo**[1,7], **Jorge Fernández**[5], **Álvaro Olivera-Nappa**[6,8], **Viola Priesemann**[2,3], and **Seba Contreras**[2,3,†*]

[1]Facultad de Medicina, Universidad de Talca, Talca, Chile.

[2]Max Planck Institute for Dynamics and Self-Organization, Göttingen, Germany.

[3]Institute for the Dynamics of Complex Systems, University of Göttingen, Göttingen, Germany.

[4]Departamento de Ingeniería en Computación, Universidad de Magallanes, Punta Arenas, Chile.

[5]Sub Department of Molecular Genetics, Institute of Public Health of Chile (ISP). Santiago, Chile.

[6]Centre for Biotechnology and Bioengineering, Universidad de Chile,Santiago, Chile.

[7]Departamento de Oncología Básico-Clínica, Facultad de Medicina, Universidad de Chile, Santiago, Chile.

[8]Department of Chemical Engineering, Biotechnology and Materials, Universidad de Chile, Santiago, Chile.

* Corresponding Author: Seba Contreras (seba.contreras@ds.mpg.de)

† These authors contributed equally

Supplementary Information

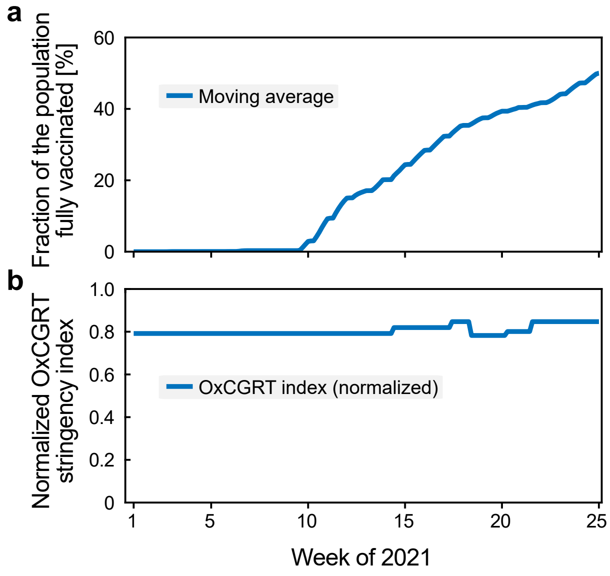

Supplementary Figure S1: **Progress of the vaccination program in Chile and the OxRCTT stringency index during vaccine rollout.**

Table S1: **Characteristic mutations in prevalent lineages.**

	Genes					
**Lineage**	**ORF1a**	**ORF1b**	**S**	**ORF3a**	**ORF8**	**N**
**B.1.1**		P314L	D614G			R203K<br>G204R
**B.1.1.348**	L1175F<br>V3718F	P314L	D614G<br>G1167A	G174D	S84L	S2Y<br>R203K<br>T366I<br>G204R
**Alpha**	T1001I<br>A1708D<br>I2230T<br>del3675/3677	P314L	del69/70<br>del144/145<br>N501Y<br>A570D<br>D614G<br>P681H<br>T716I<br>S982A<br>D1118H		Q27*<br>R52I<br>Y73C<br>S84L	D3L<br>R203K<br>G204R<br>S235F
**Lambda**	T1246I<br>P2287S<br>F2387V<br>L3201P<br>T3255I<br>G3278S<br>del3675/3677	P314L	G75V<br>T76I<br>R246N<br>del247/253<br>L452Q<br>F490S<br>D614G<br>T859N		S84L	P13L<br>R203K<br>G204R<br>G214C
**Gamma**	S1188L<br>K1795Q<br>del3675/3677	P314L<br>E1264D	L18F<br>T20N<br>P26S<br>D138Y<br>R190S<br>K417T<br>E484K<br>N501Y<br>D614G<br>H655Y<br>T1027I<br>V1176F	S253P	S84L<br>E92K	P80R<br>R203K<br>G204R

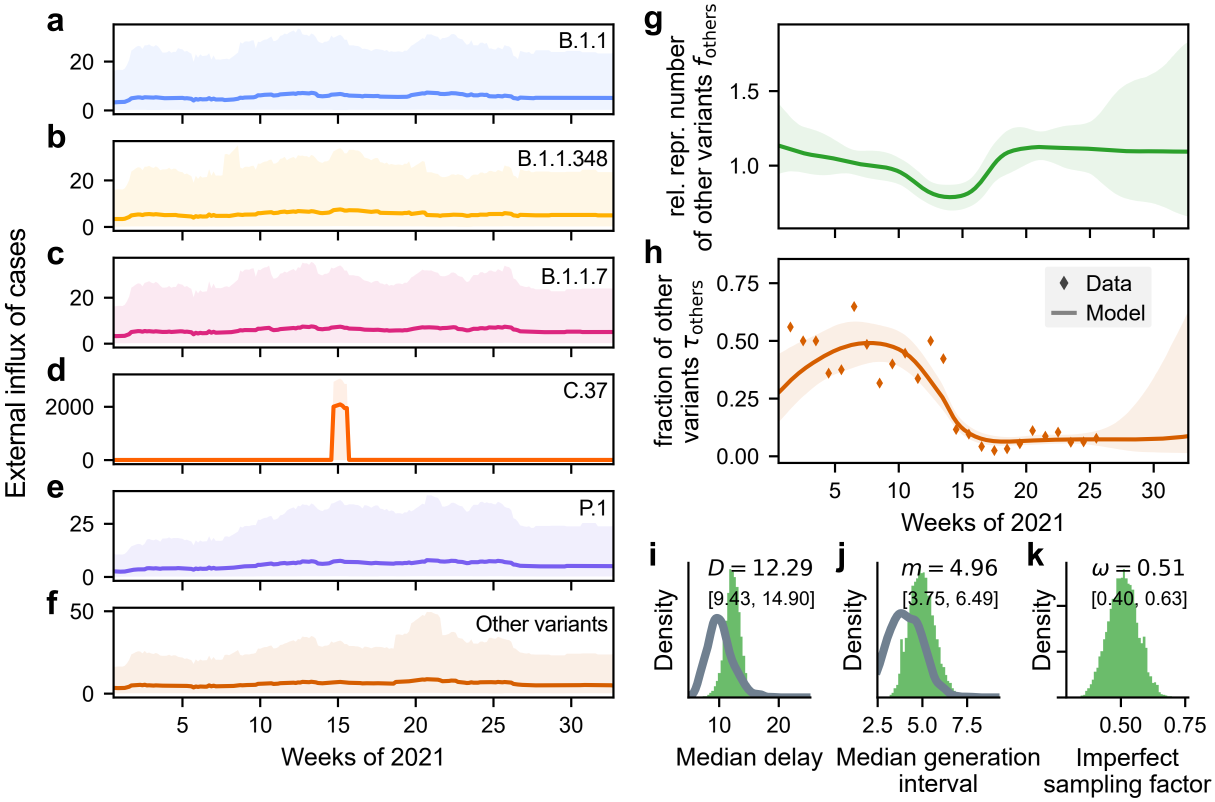

Supplementary Figure S2: **Posterior distributions of further parameters of the Bayesian model. a-f**: The external influx is low for most variants, following approximately our prior assumptions. An exception is Lambda, which eventually features a large influx when the measured fraction increases. Note, however, that the credible intervals of this influx are large, meaning that the model cannot decide whether the sudden increase in Lambda cases is due to a large influx or to a previous subsampling of Lambda cases (compare with Fig. 1 g). **g-h**: The relative reproduction number (compared to Alpha) of the modelled 'other variants' spreading in Chile. These variants are not separately modelled and, therefore, can change their relative reproduction number over time. **i-k** Prior (grey) and posterior (green) distributions of some other parameters of the model.

Table S2: **Statistical assessment of mutation enrichment in the Spike gene.**

Lineage	Levene test	Kolmogorov-Smirnov test	t-test with different variances	U-Test
Gamma	6.34e-19	0.0	0.0	0.0
Lambda	0.0*	0.0	0.0	0.0*
Alpha	5.11e-06	0.0*	0.0*	0.0*
B.1.1	8.49e-05	7.87e-05	0.0001	0.0018
B.1.1.348	0.0*	0.0*	0.0*	0.0*

[1]Values lower than 10^{-20} were considered as zero.

Table S3: **Overview of model parameters.**

Variable	Parameter
R	Effective Reproduction number
E	New infectious
S	Susceptible pool
g	Generation interval
Φ	External influx
N	Population size (19276715)
D	Delay of case detection
M	Reported (summed) cases in neighboring countries
d	Length of change point
l	Transient length of change point
Γ_c	Log-transformed reproduction number of each change point c
y	Measured number of samples sequenced
n	Total number of sequenced samples
τ	Fraction of variants in circulation
f	Contribution of variant to spread
h_w	Amplitude of weekend corrections
χ_w	Phase shift of weekend correction
Subscript v	Denotes a distinct variant
Subscript t	Denotes discretised time
Subscript c	Denotes a change poit

Table S4: **List of priors.**

Variable	Parameter
$E_{v,0}$	$\text{HalfCauchy}(\sigma = 100) \quad \forall v$
g_t	$\text{LogNormal}(t; \mu = m, \sigma = 0.4)$
m	$\text{Normal}(\mu = 4, \sigma = 1)$
f_v	$\text{LogNormal}(\mu = 0, \sigma = 1) \quad \forall v$
$\Phi_{v,t}$	$\text{HalfStudentT}_{\nu=4}(\sigma = 0.0005) \quad \forall v, t$
ω	$\text{Gamma}(\alpha = 5, \beta = 5)$
d_c	$\text{Normal}(\mu = 14c, \sigma = 5) \quad \forall c$
l_c	$\text{Normal}(\mu = 20, \sigma = 6) \quad \forall c$
Γ_c	$\text{Normal}(\mu = 0, \sigma = 0.2) + \Gamma_{c-1} \quad \forall c \neq 0$
Γ_0	$\text{Normal}(\mu = 1, \sigma = 0.2)$
κ	$\text{HalfCauchy}(\sigma = 10)$

Table S5: **Mutations becoming extinct and more predominant during vaccination roll-out in non-Spike proteins.**

**Lineage**	**Synonymous mutations becoming extinct**			**Non-synonymous mutations becoming more predominant**
**B.1.1**	E_V14V, NSP3_F106F, NSP13_L438L, NSP4_N244N			NSP13_E341D, NSP3_A231V, NSP3_A579V, NSP3_P1469S, NSP4_L438P, NSP4_T492I, NSP5_G15S, NSP6_S106-F108del, NSP8_T141M, N_P80R, N_S202T, N_S235F, ORF3a_S253P, ORF8_E92K, ORF8_Q27*
**B.1.1.348**	NSP12_N733N, NSP13_L438L, NSP3_F106F, NSP4_A416A, N_R203R, ORF3a_F43F			NSP12_I695T, NSP1_L27F, NSP3_A1215T, NSP3_K1386N, NSP3_T678I, NSP9_G38S,
**Gamma**	NSP12_D139D, NSP1_D156D, NSP3_D10D, NSP3_F106F, NSP3_P1200P, NSP3_V1298V, NSP9_Y31Y, N_R203R			None
**Alpha**	NSP12_P411P, NSP2_S36S, NSP3_F106F, NSP3_F1089F	NSP12_P411P, NSP2_S36S, NSP3_F106F, NSP3_F1089F	NSP12_P411P, NSP2_S36S, NSP3_F106F, NSP3_F1089F	NSP3_F709L, NSP6_L260F, N_R203fs,
**Lambda**	None			None

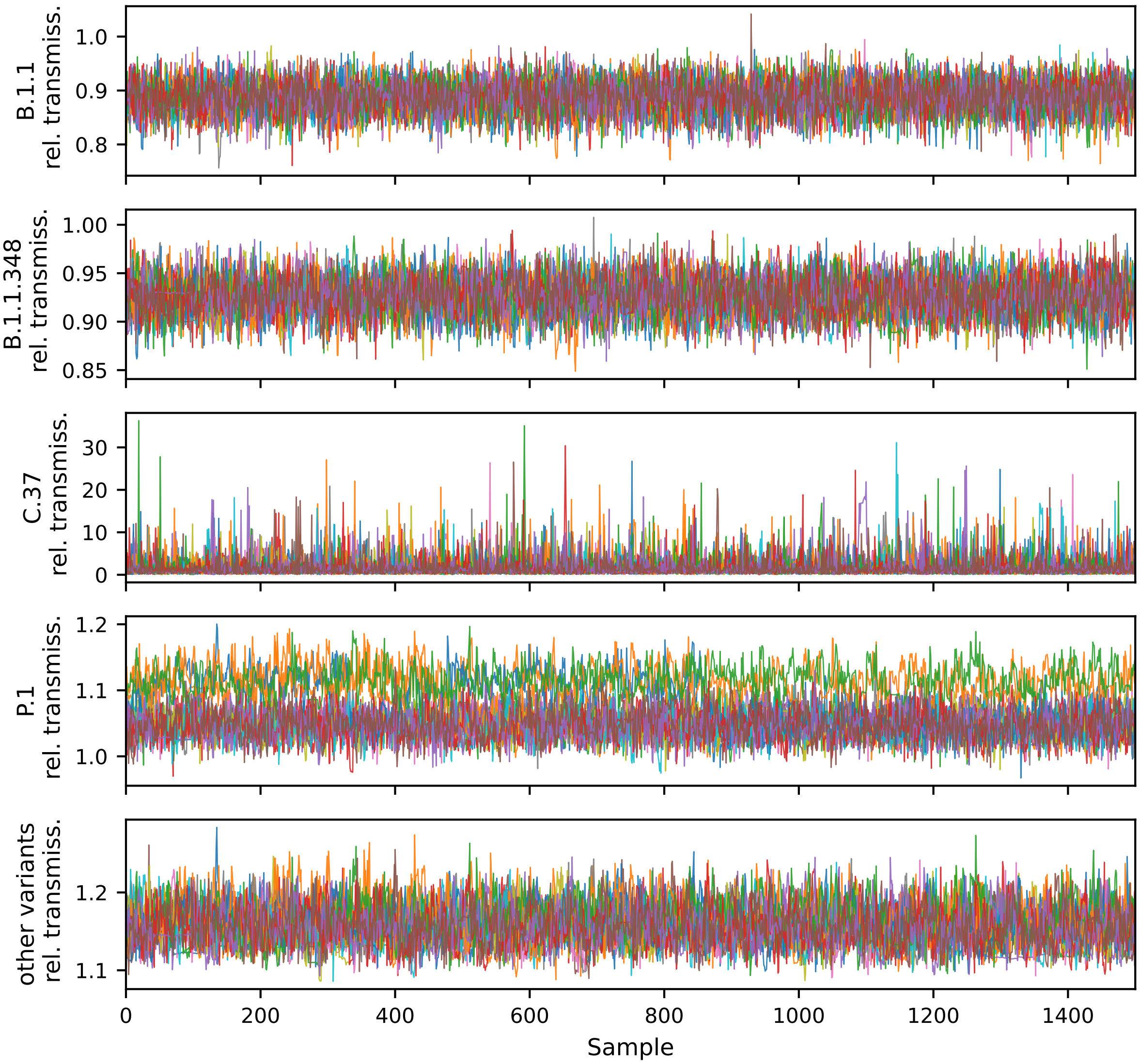

Supplementary Figure S3: **Mixing of the relative transmissibility variables**. To quantify how well the chains mix with each other, we plot here the transmissibility variables as a function of the sampling steps. Each line corresponds to another chain. According to our data, most variants mix well except Gamma (P.1), where there is a slightly bimodal solution. The peaks in the transmissibility of C.37 are a symptom that our model has difficulties explaining the sudden increase of C.37 around week 14. However, these are only individual samples that do not contribute substantially to the posterior distribution (see Fig. 1l).
